# Effect of ‘Galamsey’ on Human Fertility: A Systematic Review

**DOI:** 10.1002/hsr2.70602

**Published:** 2025-03-24

**Authors:** Samuel Kofi Arhin, Precious Barnes, Isaiah Kofi Arhin, Benedicta Owusu‐Nyarko

**Affiliations:** ^1^ School of Medical Sciences University of Cape Coast, PMB Cape Coast Ghana

**Keywords:** environmental contaminants, fertility, Galamsey, reproductive function, water pollution

## Abstract

**Background and Aims:**

In Ghana, the term ‘galamsey’ refers to illicit small‐scale gold mining. It is a primary factor in water pollution, land degradation and other environmental harm. It has also been connected to health risks like displacement and mercury poisoning. The rates of infertility among humans are rising, according to a recent study. While there are several theories explaining the rise in infertility rates, environmental contaminants may be a significant contributing role in this phenomenon. Fertility in both men and women can be impacted by a variety of environmental factors. The possible impacts of environmental contaminants on reproduction are especially concerning. We systematically reviewed the published literature on the effects of chemicals used in ‘galamsey’ on reproductive function.

**Methods:**

A comprehensive search of major databases including Medical and Allied Healthcare databases was systematically searched to identify studies published within the last period of 2010–2022. The modern SPIDER template for eligibility criteria was adopted as a guide in study selection after a literature search. Two reviewers independently screened studies for eligibility, extracted data, and assessed study quality.

**Results:**

Out of the 500 database, 20 documents were processed for their eligibility while 15 were included in the current study. The findings revealed that there is an association between the use of metals and reduced fertility in both males and females. ‘Galamsey’ exposure was also linked to decreased sperm count, motility and morphology in males as well as hormonal imbalance and increased chances of miscarriage and still birth among conceived mothers and females. Children born in these areas may have various forms of defects and malformations.

**Conclusion:**

This systematic review provides evidence that illegal mining activities have a deleterious effect on human fertility. These findings highlight the need for environmental and health reforms to address future public health threats.

## 1 Introduction

Africa holds a third of the world's natural resources and is also home to more than 2000 industrial mining operations in various phases of development [1, 2, 3]. In addition to these 2000 industrial mining operations, there are plethora of small‐scale mining activities scattered across all mining areas on the continent [2]. With the increasing need for different minerals to support a low‐carbon future, this figure is expected to rise even more by 2030 [4, 5]. The various sub‐Saharan African (SSA) nations with abundant natural resources have the potential to benefit from resource extraction. However, while the mining activities of the regulated large‐scale operations are believed to emit lower environmental hazards, that of the unregulated small‐scale mining poses diverse challenge to societies.

In Ghana, the problems caused by illegal mining popularly known as ‘Galamsey’ continue to increase due to the number of people who engage in illegal mining. It is estimated that 30,000 Ghanaians are involved in mining without license or illegal mining and this accounts for the varied challenges that mining poses to societies. These challenges, according to findings from various studies include degradation of forest reserves and vegetative cover [6, 7], destruction of farmlands and livelihood of some communities [8], destruction of water bodies [9, 10, 11] and environmental pollution [12].

While attention has been given to the extent of environmental problems that illegal mining poses, an attempt has also been made to explore health‐related consequences of illegal mining. For instance, Obiri‐Yeboah et al., [6] found that illegal mining activities result in the discharge of pollutant into rivers and water bodies which eventually creates health‐related problems to communities whose source of drinking comes from these rivers. Research studies from Emmanuel, Jerry and Dzigbodi [13], Kortei, et al., [14] and Darko, et al., [10] revealed that illegal mining causes a threat to food safety within communities where the mining activities exist. For instance, analysis from Kortei, et al., [14] revealed that toxic metals with appreciable concentrations of Cadmium, Arsenic, Lead and Mercury were found in the fish samples (*Oreochromis niloticus* and *Clarias anguillaris*) and water samples from rivers Ankobra and Pra. Darko, et al., [10] concluded that much as people within the catchment areas of these rivers consume these contaminated fishes, it poses a risk to their health. Specifically, a lifetime metal exposure evaluation based on adult and children fish consumption from the two rivers revealed carcinogenic adverse health risk to humans, although the level of health effect was not significant [10, 13].

Existing findings on health‐related consequence have shown that, indeed, activities of illegal mining pose threat to human health. However, the extent to which the illegal mining operation specifically influences the fertility or reproductive health of humans continues to remain an emerging area of research. It has been suggested that exposure of chemicals used in illegal mining has the potential of disrupting hormonal balance in both males and females, impacting fertility and reproductive health. However, since the effect of illegal mining (Galamsey) on human fertility is an emerging concept, it is important to conduct a review of the subject to understand the relationship illegal mining human reproductive health especially in Ghana.

This systematic review provides a comprehensive and up‐to‐date synthesis of the existing literature on the effect of ‘Galamsey’ on infertility, offering a novel contribution to the field in several ways:

- It identifies emerging trends and patterns in the literature, informing future research directions and policy decisions.
- It offers a nuanced understanding of the complex interactions between ‘Galamsey’ and fertility shedding new light on the current incidence and underlying mechanisms and relationships.

Overall, this systematic review provides a timely and valuable contribution to the field, advancing our understanding of ‘Galamsey’ and fertility and informing evidence‐based practice and policy.

## 2 Methods

Approach for a research study depends on several factors. While some depend on the study context (Rashid, et al. 2019), others depend on the research objectives or hypothesis (Rezigalla, 2020). However, as indicated by Panke, (2018), approach to research depends on both the study context and research objectives. In the current study, due to the large number of evidence in the field of study and within the context of the study, evidence‐based or meta‐synthesis was considered as the most appropriate research approach (Basias, and Pollalis, 2018). Furthermore, meta‐synthesis approach enables conclusions to be made on balance of evidence not on extent and quantity of literature searched but rather the quality and consistency of data in the existing literature. These published studies with varied findings make it possible to use a meta‐synthesis approach to analyze, compare and contrast findings to make informed decisions (Panke, 2018). Furthermore, the choice of this approach sets makes it easier to set the results of the study in context so that both practitioners and policy‐makers can draw valid conclusions when designing, planning and implementing useful programmes (Snyder, 2019; Hennink, et. al., 2020).

According to Greenhalgh, et al., (2018), in the midst of complex situations, it is important to include a wider range of knowledge and application of interpretation and good judgement to arrive at a meaningful conclusion. Since the concept of illegal mining and reproductive health of humans is a public health problem, it is useful to adopt a wide range of knowledge in an attempt to provide meaningful solutions. The researcher did not confine the included evidence base to reviews of Randomized Control Trials (RCTs) but rather adopted a systematic and transparent process of data synthesis to have trust in the findings. While systematic meta‐synthesis reviews are helpful in providing answers to specific, inflexible questions, exploring complicated, nuanced issues with a wealth of diverse information necessitates an interpretive, critical assessment of the larger body of available evidence.

## 3 Inclusion and Exclusion/Selection Criteria

The selection focuses on the studies that were included in this review and the basis for including them. It also discusses key concepts for the study within which the most appropriate previous studies were selected. The modern SPIDER template for eligibility criteria was adopted as a guide in study selection after a literature search (Leary and Walker, 2018). The SPIDER tool was chosen because it offers an alternative to the more frequently applied PICO (Population, Intervention, Comparison, Outcome) tool as it adapts the PICO components, making them better suited to searching for qualitative as well mixed research studies (Eriksen, and Frandsen, 2018). For instance, qualitative research ‘Outcomes’ ‘might be unobservable, or subjective constructs (for instance, perception, attitudes, opinions, awareness, views)‘ and therefore ‘Outcomes’ (O) becomes the more suitable ‘Evaluation’ (E) (Kvet, and Janáček, 2020).

## 4 Types of Studies Included

The studies that were included were studies that meet the following criteria:

- Studies that dealt with fertility of males and females within communities where illegal mining activities take place
- Studies that capture experiences of patients who are exposed to food in the form of fish or source their drinking water from rivers located in areas where activities of illegal mining exist
- Studies that focused on addressing the reproductive health‐related challenges emanating from illegal mining
- Studies that focused specifically on other health‐related problems instead of fertility‐related were excluded
- Studies that included maturity onset diabetes of youth, and gestational diabetes as well as studies, if they included only pregnant women or subjects 17 years of age or younger, were all excluded from the selected papers
- Studies that included the perspectives of only health professionals or caregivers were excluded

Various databases including Medical and Allied Healthcare databases were systematically searched to identify studies published within the last decade (2010–2022). Specifically, studies that investigated the effect of illegal mining on the fertility and/or reproductive health of humans

Medicine, Medical and Allied Healthcare Databases, Allied and Complementary Database (AMED). These were chosen due to their number of health publication which borders prevalence and causes as well as knowledge of pregnant women on anaemia. Other databases included in the study were the Cochrane Central Register of Controlled Trials (CENTRAL) and Cumulative Index to Nursing and Allied Health Literature (CINAHL) which were more fixated on healthcare procedures and clinical trials on medication and mechanisms for disease control. The focus of the current study was more on effect of illegal mining on the fertility and/or reproductive health of humans. It also focused on how chemicals used in mining like lead, mercury, arsenic and other heavy metals affect fertility. The scope of fertility includes reduced sperm quality, complications in pregnancy, altered menstrual cycle and impaired sexual health. Table 1 summaries the SPIDER procedure used in getting relevant studies from the databases.

**Table 1:** The SPIDER tool applied to the review questions.

S	Sample	People living around communities where illegal mining is rampant, as well as data on pregnant women and new born babies around communities where illegal mining
PI	Phenomenon of interest	reproductive health of males and females, neonatal diseases, sperm quality, complications in pregnancy, issues on menstrual cycle
D	Design	Published literature using quantitative, qualitative, mixed, and follow‐up studies
E	Evaluation	Perception, prevalence, views and opinions, experiences
R	Research type	Peer‐reviewed research papers, qualitative, mixed, observational, educational or academic thesis, government reports

## 5 Search Strategy

The electronic databases listed above were systematically searched using appropriate combinations of identified keywords, based on the SPIDER method and subject headings suggested by individual databases. Additional keywords were identified by examining similar review articles. Boolean phrases; AND, OR and NOT were used to combine search terms when and where appropriate (Bettany‐Saltikov and McSherry, 2016). The Boolean phrases have been summarized in Table 2.

**Table 2:** Search strategy and key words.

S	Sample	“Males” AND “Females” OR “newborns” and “above” OR AND pregnant women	
		AND	
PI	Phenomenon of interest	“ Fertility” OR Reproductive health” OR “Quality of sperm” OR “menstrual cycle”, diseases of newborn babies	
		AND	
E	Evaluation	“Perception” OR prevalence”, OR “views” OR and “opinions” OR “knowledge” OR “Experiences”

**Figure 1:**
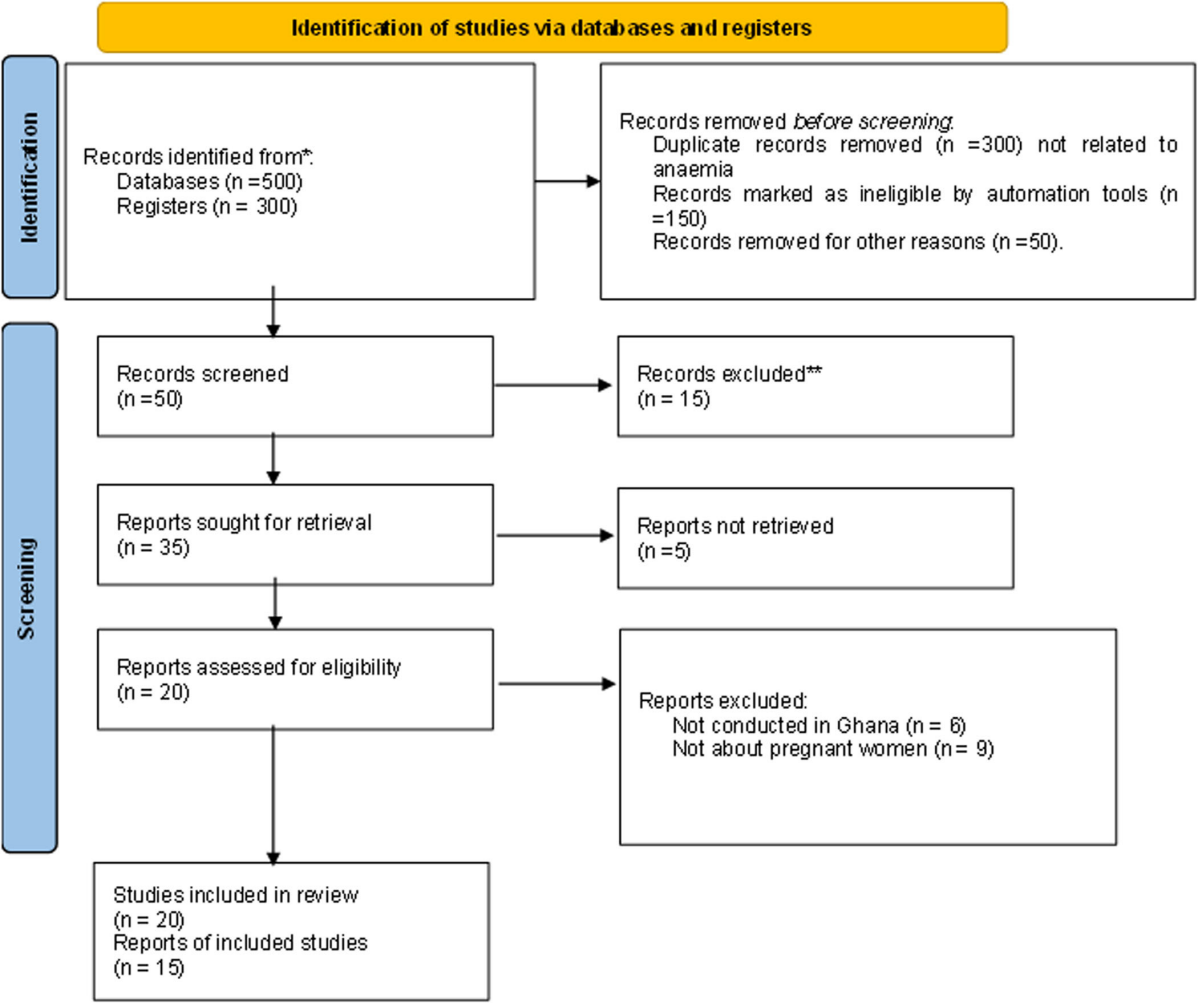
PRISMA flow chart for the identification, excluded and included studies.

In addition, reference lists (of the retrieved reviews) were hand‐searched and cross‐referenced for further studies that may have been missed. The protocol for Preferred Reporting Items for Systematic Reviews and Meta‐Analyzes (PRIMSA) was followed and this process is outlined in Figure 1.

It could be observed from Figure 1 that 500 database documents from which 300 are registered were first searched. Hundred of the documents were removed before screening and 100 were automatically removed. Additional 50 were removed for reasons such as not meeting eligibility or selection criteria. Fifty records were thereafter screened for which 10 were excluded. Twenty documents were processed for their eligibility while 15 were included in the current study.

## 6 Ethical Approval and Informed Consent

This systematic review was conducted using publicly available data and literature, and therefore did not require ethical approval or informed consent from participants. “The review was conducted in accordance with the Preferred Reporting Items for Systematic Reviews and Meta‐Analyses (PRISMA) guidelines and adhered to standard practices for systematic reviews.”

## 7 Findings

A number of reviews included in this study used quantitative and mixed approaches in conducting the research. Few studies employed the randomized controlled trial (RCT) and qualitative analysis. Due to the nature of the inclusion and exclusion criteria in the selection of studies for the current study, more of the quantitative and mixed‐method studies were included. Out of the 20 reviewed papers, 17 used the quantitative or mixed approach while three used the qualitative approach. The inclusion of more quantitative data was important to present findings that are more empirical‐based. Most qualitative studies are based on opinions and observations and hence data from qualitative studies are more subjective compared to quantitative and mixed studies (Gurevitch, et al., 2018).

Nyanza et al. (2020) focused their study on association between prenatal maternal arsenic and mercury exposure and birth outcomes in both mining and non‐mining communities. Through a longitudinal cohort analysis using 967 women from illegal mining communities and 173 women from non‐mining communities, the results revealed that half of the women (54.7%) in illegal mining communities had adverse health outcomes. Nyanza et al. (2020) further pointed out that the risk of adverse birth outcomes was significantly associated with increased prenatal exposure to arsenic and mercury. However, the number of pregnant women in non‐mining areas sampled was far below those from mining communities. Concentrating on child health, Cossa [3], found that the health of children is threatened by exposure to mining noise, air and water pollution. It was noted that children were born with various diseases including hearing and visual impairment. Although Cossa [3], did not link the physical health problems of children to reproductive issues of their parents, it was revealed that heavy metals used in the mining of gold affected both children's and parents' health.

Similarly, drawing comparison with teenage pregnant girls who are exposed to mercury for mining with their counterparts who were not exposed, Wilches‐Gutierrez [15], found that the effect of mercury was severe. Wilches‐Gutierrez [15], concluded that the ill effects of mercury on pregnant women are passed through breast milk. Therefore, babies who are exposed to mercury can have developmental mental and physical problems, from IQ loss to reduced language and memory skills. However, the impact of such mercury in breast milk which Wilches‐Gutierrez [15], claimed could affect children's IQ has not been proven through any laboratory experiment. Through a semi‐quantitative and historical analysis, N'guessan, et al. [16] found that illegal mining activities had a severe impact on the reproductive health of people living in the mining communities. In contrast to places upstream, N'guessan, et al. [16] found that the organs tested downstream incurred significant damage, as indicated by the total index. Additionally, ovarian structures appear to have a stronger impact, particularly on downstream sites, when comparing the degree of modification between the testes and ovaries. Furthermore, as the degree of harm is determined by the concentration of chemical pollutants and the duration of exposure, Cossa [3], noted that the effect of anthropogenic impact seems to be evident downstream. Changes in spermatogenic cells, liver tissues, and ovarian follicles may indicate harm to the gametogenesis process, which could eventually lower an individual's ability to procreate.

In another study, Olaolu [17], found that cadmium can disrupt steroidogenesis, postpone puberty and/or menarche, cause pregnancy loss, interfere with the menstrual cycle and reproductive hormones, cause premature births, and lower birth weights, among other impacts on female reproduction. It could be observed that unlike the predominate metals like mercury, lead and arsen which were the focus of the majority of the study, Olaolu [17], focused on cadmium Olaolu [17], contented that although there are no specific treatments for the reproductive effects of cadmium poisoning in humans, the condition can be treated with conventional medications by using metal chelators, which can be injected or taken orally. It is therefore advocated that further studies should be conducted on the potential therapeutic benefits of supplementing with antioxidants and using plants to counteract the negative effects of cadmium on living things. Pizzorno [18], also noted that toxins from mining activities if not properly handled cause fertility issues like endocrine disruption, damage to the female and male reproductive system and impaired fertility viability. Although Pizzorno [18], did not identify the nature and type of toxins, his observation is corroborated by similar studies from N'guessan, et al. [16] and Wilches‐Gutierrez [15], Van Brusselen et al. [19] assessed the possible contribution of parental and antenatal exposure to trace metals used in mining to the occurrence of visible birth defects among neonates. A case control of babies born between 2013 and 2015 was used. Specifically, newborns with visible birth defects (cases) and healthy neonates born in the same maternity ward (controls) were used the findings concluded that paternal occupational mining exposure was the factor most strongly associated with birth defects.

## 8 Discussions

The findings have revealed that although studies on health‐related problems from illegal mining have been conducted, few have actually concentrated on human fertility [15, 19]. A few have revealed that there is an association between the use of metals and the fertility of humans. It should be noted that none of these studies specifically dealt with differences in the effect between males and females. Therefore, it is difficult to indicate which gender is most affected in relation to how metals used in mining affect human fertility. Furthermore, the findings revealed that children born in places where illegal mining activities are rampant have various forms of defect. Some of these include visual and hearing impairment [17]. However, much as these defects are believed to be strongly linked with the reproductive and health conditions of their parents. It was difficult to confirm whether these defections were solely due to the presence of heavy metals in the foods of their parents.

In the various analyses and quantitative research findings, the factor most strongly linked to birth abnormalities was paternal occupational mining exposure [5, 19]. However, in the category of unclear causes of child malformation, which is more likely associated with exogenous factors, subgroup analysis revealed relevance. Thus, it appears that the various findings commonly held notion that males who work in mining are more likely to have children with malformations [2, 20]. It was further discovered that minimal to no correlation with metals in maternal blood or urine, could be explained by this paternal effect. The implication is that mine workers may bring metal‐laden dust home with them, contaminating their living space, but this ought to have been indicated by increased metal concentrations in their wives, which was not the case—at least not when the wife gave birth. It is conceivable that mutagenesis or epigenetic modifications at the spermatozoid level may have preconceptionally caused the paternal effect on their progeny. To determine if metal exposure before conception impacts gene‐specific DNA methylation profiles or other epigenetic processes and to define their impact on foetal development, more research is necessary.

Environmental metal exposure from mining continues to be a global health concern, especially in highly polluted areas, such as the African Copperbelt. Metal concentrations measured in mothers in our study were greater than previously reported in pregnant women [21]. The current study has found that there is an association between visible birth defects and paternal occupational mining exposure, and prenatal Mn and Zn concentrations in some cases albeit few [22]. These results heighten worries about the harmful effects of foetal exposure to pollution from mining. But if metals and other environmental toxins play a part in the aetiology of birth abnormalities, they will be just one element of a complicated multifactorial system.

The finding further revealed that exposure to hazardous chemicals, such as mercury and arsenic, is a serious health risk that may have a substantial impact on the health of expectant mothers and their offspring. The concept that pregnant women and their offspring are significantly at risk from illegal mining activities is supported was supported from findings in this review ([18]; Nyanza et al., al (2020). For instance, a higher prevalence of adverse birth outcomes, such as spontaneous abortion, stillbirth, preterm birth, low birth weight and visible congenital anomalies, was linked to higher exposure to arsenic and mercury during pregnancy. The risk of an adverse birth outcome was twice as high in areas with illegal mining activities as compared to areas without illegal activities.

### 8.1 Quality Assessment

The Joanna Briggs Institute Checklist for Qualitative Research was used in this review to evaluate each included paper's quality and bias risk; this tool was selected because it is specifically made for assessing congruence in systematic reviews of qualitative studies (Rezigalla, 2020). The checklist has 11 items with the emphasis on theory and method consistency, reflexivity and positionality, and ethical practise. After pilot testing, we added two fields: relevance to the synthesis, and overall quality assessment. According to Panke (2018), the study categorizes each publication into one of four groups based on the outcomes of the checklist: a key paper, a satisfactory paper, a paper that is irrelevant to the synthesis, or a manuscript that has a fatal fault. Studies won't be rejected solely on the basis of their quality, but this data will be utilized to weigh how much their findings contribute to the meta‐synthesis (Rezigalla, 2020). The study findings included an assessment of the general calibre of the examined literature, along with any research gaps or shortcomings that were found.

### 8.2 Data Extraction and Analysis

Following the Cochrane Collaboration's recommendations for the design of data collection instruments, a data extraction form was created (Bettany‐Saltikov and McSherry, 2016). To maintain consistency and flexibility in the evaluation and review of the content and quality of included studies, the form was created to reflect the key elements of the review question. In one review article, data extraction was done using separate forms for each of the publications. The form was used to collect data about the research' eligibility based on their methodologies, target populations, interventions and outcomes. The analysis was done using the thematic analysis of comparing and contrasting evidence and data from the selected studies. To effectively do this, Graebner, et al., (2023) indicated that data is to be read and reread, and this cycle is to be repeated several times to narrow down the number of codes and categorized them into identifiable themes. The current study followed the same processing to categorized findings based on themes. Five processes were followed to arrive at a specific theme. The first was familiarization where information or data from selected papers and articles were read thoroughly. The second stage involved not all relevant data in relation to the study objectives into codes. In the third stage, all similar codes were categorized into themes where these themes were reviewed in the fourth stage. The themes were finally given names as could be seen in the findings and discussions section.

## Author Contributions

**Samuel Kofi Arhin:** conceptualization, investigation, methodology, writing – review and editing, project administration, supervision. **Precious Barnes:** investigation, writing – original draft, data curation, formal analysis. **Isaiah Kofi Arhin:** methodology, investigation, writing – original draft, writing – review and editing. **Benedicta Owusu‐Nyarko:** software, formal analysis, writing – review and editing, data curation.

## Conflicts of Interest

The authors declare that they have no conflicts of interest. All authors have read and approved the final version of the manuscript. The Corresponding author, Samuel Kofi Arhin, had full access to all of the data in this study and takes complete responsibility for the integrity of the data and the accuracy of the data analysis.

## Transparency Statement

The lead author Samuel Kofi Arhin affirms that this manuscript is an honest, accurate, and transparent account of the study being reported; that no important aspects of the study have been omitted; and that any discrepancies from the study as planned (and, if relevant, registered) have been explained.

## Data Availability

The data that supports the findings of this study are available in the Supporting material of this article. The authors confirm that the data supporting the findings of this study are available within the article and its supplementary materials.
